# Fruits, Berries, and Melons

**Published:** 1872-06

**Authors:** 


### FRUITS, BERRIES, AND MELONS

Abound most and in greatest perfection in those latitudes where their peculiar agencies on the system are most essential to the preservation of human health and life. Wherever miasmas prevail, there are most indispensably needed certain qualities in whatever is adapted for the nutrition of the body which can antagonize miasmatic influences. All know that bilious diseases abound in low, flat, moist, luxurious localities, as on river bottoms, along the banks of bayous, and on undrained prairies; and these are the very places whose neighborhoods produce uncounted millions of bushels, spontaneously and in their wild state, of almost every berry that can be named, which contains that peculiar acid, so efficient in its influences on the system, as to keep it open, cool down its fevers, and enrich its blood.

THE LIVER, in its agency in purifying the blood from many of its waste, useless, and poisonous constituents, is second only to the lungs; whatever of these one leaves, the other eliminates.

A man has bilious fever; from time immemorial calomel, blue pills, or other forms of mercury, have been considered the sheet-anchor of safety, and doubtless will be till time shall be no more. It is because calomel

"ACTS ON THE LIVER,"

meaning, thereby, that in some unexplained way, either directly or indirectly, it causes that organ to do more of its appropriate work, which is to separate, to secrete from the blood many of its impure qualities, while it is passing through it to other parts of the system. At times the liver is so full of blood, so congested, that in a sense it cannot "work it up," and there it accumulates, getting more and more impure, more and more full of bile, which, instead of being yellow, is as "black as tar" in its concentration. This was found to be the condition of

DANIEL WEBSTER'S

liver after his death. His was a bilious temperament, and his habits of life were such as, combined with the temperament, were well calculated to make him bilious. But suppose in this condition a "good dose of calomel" were given a man, with ordinary vitality and strength of constitution, it would so "act on the liver," as commonly expressed, that in twelve hours after, the passages would be as black as tar, instead of a clay color, as before; and in a very few hours the patient would feel himself another man, comparatively well. Chemical research has lately ascertained, demonstrably, that the acid of fruits, in their natural state, and thus eaten, has this self-same effect on the liver; "acts" on it; makes it go to work, and separate the bile from the blood; and thus taking away the yellowness from the skin, the fever from the cheek, and the languor of disease from the eye; hence it is that in the summer and fall of the year persons who live mainly on fruits and berries and coarse breads, bread made of the whole products of the grain, are exempt from fevers, diarrhœas, and dysenteries, at the very time when whole households who eat meats and vegetables three times a day are wasting away with disease. Again, the powerful influence which berries have in the promotion of health, in averting disease, and in curing various maladies, is owing to the mechanical influence of the seeds in passing along the bowels, stimulating them to action, irritating their tender surfaces and sides, causing them to throw out water, as an irritated eye-ball "waters"; this water dissolves the hardened contents of

CONSTIPATED BOWELS, and washes them out better than any potion, pill, or purgative ever invented by the apothecary's skill. Many readers, and all educated physicians know, that white mustard seed removes costiveness when it is not desired to take medicine; dried figs are very commonly employed for the same purpose, and tomatoes also, their little seeds acting as gentle irritants in the direction named. Then, turning to

MELONS, especially the water-melon, we know the fact, but we not know the how of it, that they do act on the kidneys; they do promote free urination; and when it is remembered that almost as much of the waste of the system is carried off through the bladder as in the more solid matters, we see at once how well adapted the luscious melon is to wash out the system and make it clean and pure and healthful; all showing the wonderful wisdom and beneficence of Him of whom it is said, "His loving kindness is over all his works; in wisdom hath He made them all."

